# Detection of Tropical Fungi in Formalin-Fixed, Paraffin-Embedded Tissue: Still an Indication for Microscopy in Times of Sequence-Based Diagnosis?

**DOI:** 10.1155/2015/938721

**Published:** 2015-04-19

**Authors:** Hagen Frickmann, Ulrike Loderstaedt, Paul Racz, Klara Tenner-Racz, Petra Eggert, Alexandra Haeupler, Ralf Bialek, Ralf Matthias Hagen

**Affiliations:** ^1^Department of Tropical Medicine at the Bernhard Nocht Institute, German Armed Forces Hospital of Hamburg, Bernhard-Nocht Street 74, 20359 Hamburg, Germany; ^2^Institute for Microbiology, Virology and Hygiene, University Medicine Rostock, Schillingallee 70, 18057 Rostock, Germany; ^3^Central Laboratory Department/Department of Clinical Chemistry, University Medicine Goettingen, Robert Koch Street 40, 37075 Göttingen, Germany; ^4^Department of Infectious Disease Pathology, Bernhard Nocht Institute for Tropical Medicine Hamburg, Bernhard Nocht Street 74, 20359 Hamburg, Germany; ^5^LADR GmbH MVZ Dr. Kramer & Kollegen, Lauenburger Straße 67, 21502 Geesthacht, Germany

## Abstract

*Introduction*. The aim of the study was the evaluation of panfungal PCR protocols with subsequent sequence analysis for the diagnostic identification of invasive mycoses in formalin-fixed, paraffin-embedded tissue samples with rare tropical mycoses. *Materials and Methods*. Five different previously described panfungal PCR/sequencing protocols targeting 18S and 28S ribosomal RNA gene fragments as well as internal transcribed spacer 1 and 2 fragments were evaluated with a collection of 17 formalin-fixed, paraffin-embedded tissue samples of patients with rare and/or tropical invasive mycoses, comprising chromoblastomycosis, coccidioidomycosis, cryptococcosis, histoplasmosis, mucormycosis, mycetoma/maduromycosis, and rhinosporidiosis, in a proof-of-principle analysis. *Results*. The primers of the panfungal PCRs readily and predominantly reacted with contaminating environmental fungi that had deposited on the paraffin blocks. Altogether three sequence results of histoplasmosis and mycetoma samples that matched the histological assessment were associated with sample age <10 years and virtually without PCR inhibition. *Conclusions*. The high risk of amplifying environmental contaminants severely reduces the usefulness of the assessed panfungal PCR/sequencing protocols for the identification of rare and/or tropical mycoses in stored formalin-fixed, paraffin-embedded tissues. Histological assessment remains valuable for such indications if cultural differentiation is impossible from inactivated sample material.

## 1. Introduction

Invasive mycoses are rare but associated with severe diseases with high mortality [1, 2]. Accordingly, reliable diagnostic approaches are necessary. Cultural growth with subsequent identification and antifungal susceptibility testing is desirable whenever possible as a diagnostic gold standard [3]. However, cultural growth of fungi is time-consuming and, as with, for example,* Loboa loboi* (causative agent of lobomycosis),* Pneumocystis jirovecii*, or* Rhinosporidium seeberi* (causative agent of rhinosporidiosis) [3–6], is not always possible. The time frame within which cultural results may be expected ranges from 1 day for very rapidly growing fungi such as Mucorales up to 6 weeks for slowly growing pathogens such as* Histoplasma* spp. or* Paracoccidioides* spp. [3]. Cultural growth of particularly harmful fungal pathogens such as* Histoplasma capsulatum*, which can cause invasive disease even in the immunocompetent human host and are readily transmitted under laboratory conditions [7], has to be performed under biosafety level 3 (BSL-3) conditions.

However, culture will fail if potential microbial causes of the pathological findings are not considered at the time of sample acquisition, so formalin-fixed, paraffin-embedded bioptic material is collected for subsequent histological assessment. After formalin-based sample inactivation, histological assessment is usually the method of choice in the diagnostic algorithm. Nevertheless, a recent 10-year single-center study impressively demonstrated an analytical correctness of no more than 79% of histological findings for assessed invasive mycoses [3]. Accordingly, preliminary histological results should be interpreted with care [8] and supported by cultural approaches whenever possible.

If culture is not possible for logistic, technical, or other reasons and if there is no alternative sample material available other than the formalin-fixed, paraffin-embedded material, PCR-based diagnostic approaches may be considered. Various PCR protocols for the specific detection of certain invasively growing fungi with human pathogenic potential, for example, Mucorales, in tissue have been introduced [9–15]. There are even published multicenter evaluations for PCR-based identification of invasive candidosis or aspergillosis [16]. Best results can be expected for short-fragment PCRs, because formalin-induced cross-linking between DNA strands or DNA–protein cross-links can inhibit the amplification of longer fragments. Such random events occur approximately every 1000 base-pairs, reducing the chances of long-fragment amplifications, particularly with low amounts of target DNA [17–20]. However, the use of genus- or species-specific PCRs requires prior clinical suspicion, because primers and probes will bind only to their specific target organisms. Further, the sensitivity of PCR of formalin-fixed, paraffin-embedded tissues will decrease with low parasite density and greater sample age, as recently demonstrated for invasive amebiasis samples by our group [20].

If there is no explicit suspicion regarding the potential fungal pathogen, broad-range PCRs with consecutive sequence-based analysis have to be performed, usually amplifying longer DNA fragments of several hundred base pairs. Numerous protocols have been described for the sequence-based identification of fungi in formalin-fixed, paraffin-embedded tissue, usually targeting the 18S rRNA gene, the 28S rRNA gene, or ITS (internal transcribed spacer) fragments [10, 16, 21–27]. These techniques are usually applied in industrialized, mostly Western, countries where diagnostic Sanger sequencing is readily available. Accordingly, they are predominantly evaluated with bioptic samples of patients with invasive mycoses that are typical for that part of the world, that is, invasive candidiasis, invasive aspergillosis, or, to a lesser degree, mucormycosis [21, 25, 27]. For other invasive fungi, in particular tropical fungi, evaluation data are scarce.

In this study, we evaluated five published PCR/sequencing protocols for the identification of fungal pathogens in formalin-fixed, paraffin-embedded bioptic tissue samples [22–27] with samples of patients with rare and tropical mycoses from the Department of Infectious Disease Pathology at the German National Reference Centre for Tropical Diseases Bernhard Nocht Institute. The tissues were up to 30 years old and contained low concentrations of pathogens as assessed by microscopic analysis. This proof-of-principle assessment aimed to answer the questions whether the described diagnostic protocols provide reliable results with less usual fungal pathogens in complex sample matrices and whether there is still a role for microscopy in such instances.

## 2. Materials and Methods

### 2.1. Specimen Collection

In a 30-year period between 1984 and 2013, a specimen collection comprising a total of 17 paraffin-embedded, buffered formalin-fixed bioptic samples demonstrating histologically confirmed invasive mycosis due to tropical or other rare fungal pathogens was established at the Department of Infectious Disease Pathology of the Bernhard Nocht Institute for Tropical Medicine in Hamburg, Germany. Histological diagnoses comprised chromoblastomycosis (*n* = 3), coccidioidomycosis (*n* = 2), histoplasmosis (*n* = 4), histoplasmosis or cryptococcosis (*n* = 1), mucormycosis (*n* = 2), mycetoma/maduromycosis (*n* = 3), and rhinosporidiosis (*n* = 2). Culture-based diagnostic results were not available. The sample collection included lymphatic node tissue (*n* = 2), skin biopsy (*n* = 5), a biopsy of a nasal polyp (*n* = 1), vulva exudate (*n* = 1), lung tissue (*n* = 3), bone, muscular tissue, and connective tissue from the spine extension of the third thoracic vertebra (*n* = 1), tissue from the tricuspid valve (*n* = 1), bioptic material from the bottom lip (*n* = 1), material from the ethmoid (*n* = 1), and a biopsy from a wound of the foot (*n* = 1). The ages of the samples ranged from 1 to 30 years at the time of DNA extraction, with a median of 11 years in a left-shifted distribution (Table 1).

To exclude microtome-associated contamination, three randomly chosen, recently obtained bioptic samples from patients with diseases other than invasive mycosis were included in the analyses as negative controls. As invasive mycosis is a rare diagnosis [1, 2], the risk of the negative control patients being infected with invasively growing fungi can be considered extremely low.

### 2.2. Sample Preparation

The small quantities of residual material from the tissue blocks in the specimen collection allowed for only one mode of DNA preparation, which has been reported to be optimal for the recovery of fungal DNA from formalin-fixed, paraffin-embedded tissues [26], with minor modifications. One 25 *μ*m thick section was obtained from each paraffin block, including the blocks with the negative control tissues. Weighting of the samples was not performed. Instead of this, DNA amounts within the samples were measured as detailed below. The negative control samples were distributed among the tissue blocks and each section was cut at a different position of the disposable knife of the microtome. If all samples had been cut at the same position of the disposable knife, this might have allowed for DNA cross-contamination due to attached cells at that position of the knife from other, previously cut tissues. For deparaffinization, the sections were put into 1.5 mL tubes and exposed for 2 × 10 minutes to 1200 *μ*L xylene and for 3 × 10 minutes to 1200 *μ*L 100% ethanol under gentle constant agitation. Following each 10-minute step, the supernatant was discarded after centrifugation for 10 minutes at 13 000 ×g. Finally, the samples were air-dried. The further DNA extraction was performed according to the manufacturer's instructions for the DNA FFPE tissue kit (Qiagen, Hilden, Germany) with the following modifications. Proteinase K digestion at 56°C was performed overnight until the suspension was clear. In line with the described protocol [26], fungal cell walls in the pellet were lysed with 400 units of* Arthrobacter luteus* lyticase L2524 (Sigma-Aldrich Corporation, St. Louis, MO, USA; a surrogate product for lyticase L4276 (Sigma-Aldrich Corporation) which was no longer available) per sample for 45 minutes at 37°C. The rest of the DNA extraction was performed exactly as described by the manufacturer of the DNA FFPE tissue kit. Subsequently, the material was used for PCR.

In addition to thick sections for PCR, thinner 4–6 *μ*m sections were obtained to histologically confirm and, in case of* Histoplasma capsulatum* detection, semiquantify the presence of fungal pathogens in tissue by classical hematoxylin and eosin (HE), Giemsa, periodic acid-Schiff (PAS), and Grocott staining.

### 2.3. Molecular Diagnostic Approaches

#### 2.3.1. Sample Quality Assessment and Inhibition Control PCR

The DNA amount in the samples was quantified using a Pico 100 Picodrop Microliter Spectrophotometer (Picodrop Ltd., Hinxton, UK) according to the manufacturer's instructions. DNA quality was assessed in all samples by a Taqman PCR targeting a 155-base-pair fragment of the human 18S rRNA gene as previously described [12] with minor modifications (Table 2).

Plasmids containing phocid herpesvirus 1 (PhHV-1) sequences were added to each sample as internal controls to exclude sample inhibition. Dilution was chosen to achieve cycle threshold- (Ct-) values of about 18. Primers and probes were used as previously described [28] for the amplification of an 89-base-pair fragment with slight modifications (Supplementary Material 1).

#### 2.3.2. Specific PCR for* Histoplasma* spp. and Mucorales with Consecutive Sequencing

Samples with histological suspicion of histoplasmosis or Mucorales infection were subjected to specific PCRs with consecutive sequencing for confirmatory testing. Nested PCR for histoplasmosis targeting the gene encoding the unique fungal 100-kDa-like protein [29] and seminested PCR for Mucorales [30] were applied as described. Only cases with positive results were considered as confirmed infections. In case of negative PCR results, cases were defined as “histologically suspected”* Histoplasma* or Mucorales infections.

#### 2.3.3. Panfungal PCRs

A total of five panfungal PCRs that have been evaluated for the diagnostic identification of invasive mycoses, including in some cases from formalin-fixed, paraffin-embedded tissue [22–27], were applied; in the following these are referred to as PCR 1 to PCR 5. The PCR panel comprised three real-time PCRs and two traditional block cycler PCRs. The PCR targets comprised fragments of the 18S rRNA gene (PCR 1, according to [22, 23], applied on a LightCycler 2.0 (Roche, Basel, Switzerland)) and the 28S rRNA gene (PCR 3, according to [24, 27], applied on a Corbett RotorGene 6000 (Qiagen, Hilden, Germany)) as well as the internal transcribed spacer (ITS) regions ITS-1 (PCR 5, according to [25], applied on a TProfessional Basis cycler (BioMetra, An Analytik Jena Company, Jena, Germany)) and ITS-2 (PCR 2, according to [24, 27], applied on a Corbett RotorGene 6000, and PCR 4, according to [26], applied on a TProfessional Basis cycler) with species-dependent variable fragment lengths between 200 and 500 base pairs [22–27]. All PCR protocols were adapted to the cyclers that were used prior to the analysis. The respective adaptations were cycler-specific and comprised reaction mixes, MgCl_2_ contents, optimal primer-probe-concentrations, and cycling protocols. Presentation in all details can be found in supplementary material 1 (see Supplementary Material available online at http://dx.doi.org/10.1155/2015/938721) of this paper.

DNA of clinical* Candida glabrata* and* Candida tropicalis* isolates was used as positive controls. Water served as a negative control. The bands of the traditional block cycler PCRs were visualized using a FlashGel System (Lonza, Basel, Switzerland) according to the manufacturer's instructions. Amplicons of positive PCRs were purified using the NAT Clean-up/Nucleospin Extrakt II kit (Macherey & Nagel, Düren, Germany) according to the manufacturer's instructions and sent for Sanger sequencing to SeqLab GmbH (Göttingen, Germany). Sequences from the ab1-files obtained were aligned using BioNumerics 7.1 software (Applied Maths, Sint-Martens-Latem, Belgium). The alignment settings were open gap penalty 100%, unit gap penalty 0%, match score 100%, and fast algorithm (= minimum match sequence 2, maximum number of 98).

The sequences obtained were compared with deposited sequence information using the BLAST algorithm (http://blast.ncbi.nlm.nih.gov/Blast.cgi/) excluding human and model sequences and restricting the displayed results to fungal sequences. Best matches regarding both coverage and sequence identity were considered. To evaluate the quality of the results, the criteria suggested by the CLSI (Clinical and Laboratory Standards Institute) guideline MM18-A “Interpretive Criteria for Identification of Bacteria and Fungi by DNA Target Sequencing; Approved Guideline” [31] were employed. In detail, ≥99% identity was demanded for identification at species level, ≥97% identity for identification at genus level. Further, 0.8% separation between different species was demanded for a reliable discrimination. Deposition of obtained sequences to databases was not intended and was not carried out because no unambiguous diagnostic gold standard was available with which to confirm the fungal species identity.

### 2.4. Microscopic Assessment

**Figure 1:**
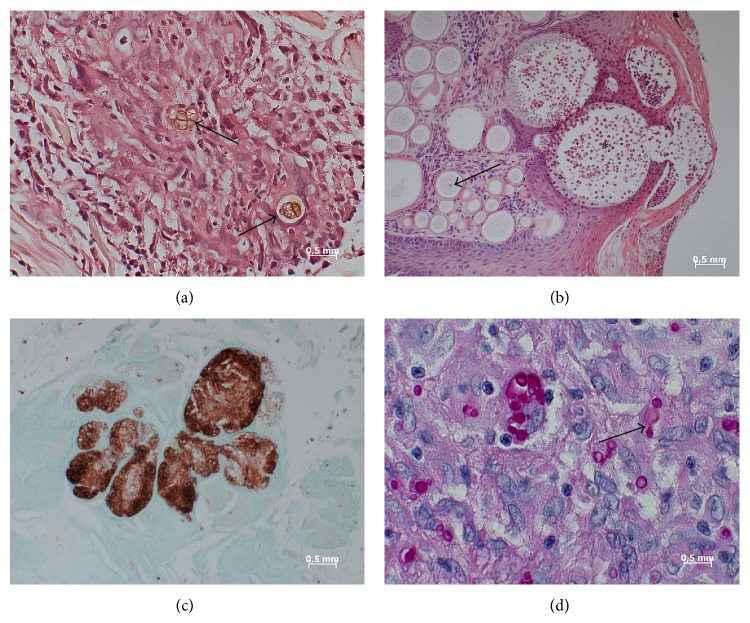
Photographs of representative fungi-containing samples analyzed in this study. (a) Case 1. Chromoblastomycosis of the skin. Fungal elements can be recognized in HE-stained sections as brownish-yellow pigmented bodies (arrows). A separation of the thick-walled fungus is also visible (multiform body). (b) Case 9. Skin lesion in rhinosporidiosis. Numerous spherical structures varying in diameter in an age-dependent manner. Immature forms (trophocytes) contain nucleus (arrow) and cytoplasm. Mature forms (sporangia) contain numerous endospores (asterisk). The rupture of this form is also visible. HE staining. (c) Case 10. Mycetoma. Lobulated grain with light colored center (brown) in lung. Grocott stain. (d) Case 11.* Histoplasma* infection. The periodic acid-Schiff reaction reveals many ellipsoidal yeast cells (red) inside macrophages and a giant cell. The arrow shows a budding.

Thin 4–6 *μ*m neighboring sections that were cut in addition to the thick sections for PCR were analyzed by microscopy of HE-, Giemsa-, PAS-, and Grocott-stained slides by an experienced pathologist. Assessment of fungal elements was done using a Zeiss AxioImager M1 microscope equipped with an AxioCam MRc5 digital camera and AxioVisionRel 4.6 software (Zeiss, Jena, Germany). Briefly, nonoverlapping digital images (160 *μ*m × 210 *μ*m) were captured using a ×40 objective lens. Illustrations of representative mycosis samples are shown in Figure 1. In case of histological suspicion of* Histoplasma capsulatum* infection, semiquantification was performed. To do so, the numbers of pathogens per unit area (160 *μ*m × 210 *μ*m) were manually counted and averaged. In 4 out of 5 samples, 15 unit areas were assessed. The smaller size of the fifth sample allowed for the assessment of 13 unit areas only.

### 2.5. Ethics

Ethical clearance for the anonymous retrospective molecular assessment of residual materials from formalin-fixed, paraffin-embedded tissues for evaluation purposes was obtained from the Ethics Committee of the Medical Association of Hamburg, Germany (document number WF-028/13).

## 3. Results

### 3.1. Morphological Sample Characteristics

A total of 17 formalin-fixed, paraffin-embedded tissue specimens from patients with histologically confirmed invasive mycoses, deposited for 1 to 30 years (mean 11.5 years), were included in the analysis. Microscopic assessment allowed the detection of fungal elements on microscopic slides of all mycosis samples. The fungal density varied substantially from slide to slide owing to inhomogeneous distribution of the pathogens in tissue. Furthermore, in the case of filamentous fungi, elements of a multiply-cut filament were indistinguishable from single cuts of multiple filaments. For these reasons, semiquantification of fungi per unit area (160 *μ*m × 210 *μ*m) was abandoned as unreliable. In case of histological suspicion of nonfilamentous* Histoplasma capsulatum*, semiquantification was performed. An average of < 10 pathogens per sample unit was observed for all 5 samples, ranging from 2 to 9 fungal elements per sample unit.

### 3.2. Sample Quality Assessment

The mean DNA-amount in the fungal samples was 123.6 ng/*μ*L, ranging from 12.3 to 618.9 ng/*μ*L. In the fresh negative control material, higher DNA amounts were measured with a mean value of 647.2 ng/*μ*L, ranging from 609.7 to 676.6 ng/*μ*L (Table 2).

Positive results of the DNA-quality assessment 18S rRNA gene PCR were obtained for all but one sample. However, Ct-values were distributed over a wide range from 14 to 33. Ct-values did not seem to be influenced by sample age alone, as there was also a 17-year-old sample with a Ct-value as low as 15 (Table 2). The variety of sample materials (Table 1) made a direct comparison difficult, however.

Inhibition control PCR was positive for all analyzed samples. However, Ct-values ranged from 18 to 26, corresponding to low to moderate sample inhibition up to about 2.5 decadic logarithmic steps. Again, there was no obvious association between sample age and sample inhibition (Tables 1 and 2).

### 3.3. Results of Specific PCRs with Consecutive Sequencing

Five samples with histological suspicion of histoplasmosis and two samples with histological suspicion of Mucorales infections were subjected to specific PCR with consecutive sequencing. Only one out of five suspected histoplasmosis cases and one out of two suspected Mucorales infections could be confirmed by this approach (Table 2). Consequently, the remaining 5 unconfirmed cases were considered as histologically suspected only.

### 3.4. PCR/Sequencing Results in relation to Their Potential Predictors

The panfungal PCRs readily reacted with DNA from contaminating spores of environmental fungi, even in the negative control materials, as demonstrated by the sequencing results (Table 3). Details on PCR and sequencing results, comprising cycle threshold (Ct) values and peaks of the melting curve analyses, sequence fragment lengths as well as sequence identity, and coverage percentages, are presented in the supplementary materials 2–6 of this paper. In repeated instances (Table 3), sequence quality was noninterpretably poor, mostly due to overlapping sequences of different fungal species, when the used panfungal primers also reacted with fungal DNA of contaminants from the environment. Depending on the panfungal PCR used, sequence fragments of different fungal species were obtained from the same sample in several instances (Table 3). Primers of each PCR thus preferentially reacted with certain contaminants that differed from one protocol to another. Only in one case of histologically suspected histoplasmosis affecting cardiac valve material did all positive PCRs with interpretable sequence results suggest* Candida parapsilosis*, suggesting histological confusion of invasive candidiasis with histoplasmosis [8] in this instance.

Sequences of the microscopically observed pathogens, however, were not detected with the exception of three instances. Panfungal PCRs 1 and 4 identified* Histoplasma capsulatum* sequence fragments in bioptic material from the patient's bottom lip (Table 3). Of note, this was the only histoplasmosis case that was confirmed by specific PCR (Table 2). For PCR 1, however, sequence quality was unacceptably low with only 92% sequence identity and coverage of only 71% (supplementary material 2). Further, PCR 4 (Table 3) allowed the identification of* Madurella mycetomatis* from a biopsy of a wound on the foot of a mycetoma patient.

All sample materials with correct species identifications were younger than 10 years of age. Further, no significant PCR inhibition was detectable for the respective samples; the Ct-values were ≤20. Of note is that the missed coccidioidomycosis and chromoblastomycosis cases that were younger than 10 years all showed Ct-values ≥23 in the inhibition control PCR (Table 2). However, even in young samples without detectable relevant inhibition, not all PCR and sequencing approaches led to correct results as shown for the histoplasmosis sample from the bottom lip (Table 3). The number of correct sequence-based identifications was too low to allow a statistical assessment of favorable factors.

With the exceptions of panfungal PCRs 1 and 4, the other panfungal PCRs led to a varying number of failed reactions in the 17 microscopically positive samples assessed, that is, one failure for PCR 2, three failures for PCR 3, and as many as seven failures for PCR 5. In contrast, PCRs 1–4 showed positive results with negative control samples, while only PCR 5 was not associated with such false-positive reactions (Table 3). For PCR 1, Ct-value assessment against background fluorescence was impossible for unknown reasons, allowing the identification of positive test results only by melting curve analysis.

## 4. Discussion

In addition to the requirement for the presence of a critical number of pathogens in the analyzed materials, the histological identification of pathological fungi in tissue sections of patients with invasive mycosis demands specific skills and a high degree of experience, as suggested by the considerable number of mismatches in comparison with cultural diagnostic approaches [3, 8]. In line with these previous results, only one out of five histologically suspected histoplasmosis cases and one out of two suspected Mucorales infections could be confirmed by specific PCR in the approach described herein.

Molecular tests such as panfungal PCR with subsequent sequence analysis might help pathologists confirm the differential diagnosis, but nonspecies-specific diagnostic approaches are easily disturbed by environmental contamination, for example, located within the paraffin, unless protective measures are assured. Species-specific simplex or multiplex PCRs [9–16] might be an alternative. Other than panfungal PCRs with subsequent sequence analysis, however, such species-specific assays require a specific suspicion regarding the potential etiological agent for their selection.

Here we assessed the performance of five panfungal PCRs with subsequent sequence analysis that were designed for the diagnosis of invasive mycosis partly from rather difficult material such as formalin-fixed, paraffin-embedded tissues and evaluated in Western industrialized settings [22–27] for the diagnosis of rare and/or tropical invasive mycoses, making use of a collection of 17 samples from the German National Reference Center for Tropical Infections. All samples were originally obtained from patients with confirmed invasive rare and/or tropical mycosis and were analyzed by experienced pathologists.

Some of the assessed panfungal PCR and sequencing protocols showed promising evaluation results in previous studies [25–27] with invasive mycoses due to* Candida* spp. or* Aspergillus* spp. even from formalin-fixed, paraffin-embedded tissue samples after appropriate deparaffinization. The results presented here demonstrate, however, that panfungal PCRs from tissues in long-term stored paraffin blocks are generally prone to reactions with fungal spore contaminants, at least if no special preventive measures against contamination are employed. Contamination of the tissue-containing paraffin blocks with spores of environmental fungi was highly likely, as the blocks were stored for several years without particular precautions against deposition of spores. Contamination of paraffin with fungal spores might be an alternative explanation. Interestingly, the primers reacted more readily with DNA of environmental contaminants than with the rare and/or tropical fungi in our sample collection, thus considerably reducing the value of the protocols for use in cases of suspicion of tropical mycoses. Even more relevantly, positive PCR results occurred in negative control samples and failed PCR was infrequently observed in microscopically positive samples. Accordingly, PCR did not even reliably discriminate invasive mycosis from nonmycotic disease. Sample age and PCR inhibition seemed to be relevant factors here.

According to our analysis, the few correct sequencing results were observed in comparatively fresh samples that were younger than 10 years of age and that did not show considerable PCR inhibition. Even moderate inhibition prevented the amplification and subsequent sequencing of DNA fragments of the microscopically observed invasive fungal pathogens. The reliability of PCR and sequencing seems to be affected by sample quality and sample storage time. While 20 years of storage time marked the cut-off for diagnostic reliability of target-specific PCR as observed in a previous study [20], this time limit is obviously even shorter in the case of panpathogen PCRs with subsequent sequencing. Notably, the influence of storage time on the quality of DNA was not directly reflected in the semiquantitative results of a PCR targeting a 155-base-pair fragment of the human 18S rRNA gene owing to the variety of different sample materials and degrees of PCR inhibition. This variety limits the interpretability of the results of this proof-of-principle assessment with rare and/or tropical causes of invasive mycosis.

As in recent reports on fungal specimens [26], the mode of sample preparation was in principle effective in releasing fungal DNA and allowing its amplification even in samples that had been stored for years. However, PCR inhibition remained considerable in several samples. Alternative protocols for DNA preparation from formalin-fixed, paraffin-embedded tissue [26] might have led to an increased yield of fungal DNA from the microscopically observed pathogens but could not be assessed as the low quantities of residual material did not allow for comparative testing of different preparation schemes, an admitted limitation of the study.

Panfungal PCR with subsequent sequencing clearly performed less well than classical microscopy of stained sections for the identification of invasive rare and/or tropical fungal infections, at least when histology was performed by experienced pathologists. Matching sequence results were observed only in individual instances, underlining the importance of preserving histological skills in diagnostic routine. As suggested by the results of specific PCR analyses with the samples of histologically suspected histoplasmosis and mucormycosis as well as by previous studies [3, 8], however, specificity of microscopy shows considerable limitations as well.

## 5. Conclusion

We conclude that sequence results obtained after panfungal PCR can only be considered as confirmatory information in case of matching with preliminary histological results owing to the high risk of contamination of paraffin blocks with environmental fungal spores. Other histological techniques such as immunohistochemistry [8, 32] and* in situ* hybridization [8, 33] or, alternatively, specific PCRs for formalin-fixed, paraffin-embedded tissues [8, 29] can be used for the confirmation of diagnosis if mycological culture is not possible. Preliminary histological results should be interpreted with care and supported by cultural approaches whenever possible.

If mycological culture is possible, it is the reference method for the diagnosis of mycosis. In many mycoses, histology only allows a presumptive diagnosis that needs confirmation by mycological culture [8].

## Supplementary Material

Altogether, 6 supplementary materials are presented to provide further details regarding the applied methods and the observed results.
Supplementary material 1 describes the applied pan-fungal PCR protocols, the inhibition control PCR and the sample quality assessment PCR in detail.
The supplementary materials 2-6 are an addition to table 3 of the paper. They provide more detailed information regarding the observed ct-values, melting curve peaks (if applicable), detection of visible bands in electrophoresis (if applicable), readable sequence fragment lengths, coverage and sequence identities of pan-fungal PCRs 1-5. Thereby, they allow for a better interpretation of PCR and sequencing results.

## Figures and Tables

**Table 1:** Histological diagnosis, material, and age of the 17 analyzed samples from patients with invasive mycosis.

Sample I.D.	Histological diagnosis	Sample material	Sample age (years)
Case 1	Chromoblastomycosis	Skin biopsy	30
Case 2	Mucormycosis	Material from the ethmoid	17
Case 3	Histoplasmosis	Lymph node biopsy	15
Case 4	Mucormycosis	Skin biopsy	14
Case 5	Histoplasmosis or cryptococcosis	Lymph node biopsy	13
Case 6	Chromoblastomycosis	Skin biopsy	12
Case 7	Rhinosporidiosis	Biopsy of a nasal polyp	12
Case 8	Mycetoma/maduromycosis	Vulva exudate	11
Case 9	Rhinosporidiosis	Skin biopsy	11
Case 10	Mycetoma/maduromycosis	Lung tissue	11
Case 11	Histoplasmosis	Bone, muscular tissue, and connective tissue from the spine extension of third thoracic vertebra	11
Case 12	Histoplasmosis	Tissue from the tricuspid valve	11
Case 13	Chromoblastomycosis	Skin biopsy	8
Case 14	Histoplasmosis	Material from the bottom lip	8
Case 15	Coccidioidomycosis	Lung tissue	6
Case 16	Coccidioidomycosis	Lung tissue	5
Case 17	Mycetoma/maduromycosis	Biopsy from a wound of the foot	1

**Table 2:** Results of inhibition control PCR (phocid herpesvirus PCR), DNA extraction control PCR (18S rRNA gene PCR), and specific PCR/sequencing results for *Histoplasma capsulatum* and Mucorales. Ct = cycle threshold. N.a. = not applicable.

Sample I.D.	Histological diagnosis	Ct-value of phocid herpesvirus PCR	Ct-value of human 18S rRNA gene PCR	DNA concentration as measured by Picodrop	*Histoplasma*-specific nested PCR (and consecutive sequencing)	Mucorales-specific seminested PCR (and consecutive sequencing)
Case 1	Chromoblastomycosis	22	23	12.3 ng/*µ*L	N.a.	N.a.
Case 2	Mucormycosis	18	15	14.6 ng/*µ*L	Negative	Negative
Case 3	Histoplasmosis	18	14	246.3 ng/*µ*L	Negative	Negative
Case 4	Mucormycosis	20	—	26.9 ng/*µ*L	Negative	Positive (100% *Lichtheimia*/*Absidia corymbifera*)
Case 5	Histoplasmosis or Cryptococcosis	20	19	157.6 ng/*µ*L	Negative	Negative
Case 6	Chromoblastomycosis	26	30	414.5 ng/*µ*L	N.a.	N.a.
Case 7	Rhinosporidiosis	26	23	62.7 ng/*µ*L	N.a.	N.a.
Case 8	Mycetoma/maduromycosis	22	33	618.9 ng/*µ*L	N.a.	N.a.
Case 9	Rhinosporidiosis	18	22	21.9 ng/*µ*L	N.a.	N.a.
Case 10	Mycetoma/maduromycosis	25	15	16.8 ng/*µ*L	N.a.	N.a.
Case 11	Histoplasmosis	21	30	104.6 ng/*µ*L	Negative	Negative
Case 12	Histoplasmosis	24	15	44.3 ng/*µ*L	Negative	Positive (no interpretable sequence results due to poor sequence quality)
Case 13	Chromoblastomycosis	23	14	33.8 ng/*µ*L	N.a.	N.a.
Case 14	Histoplasmosis	20	17	175.5 ng/*µ*L	Positive (99% *Histoplasma capsulatum*)	Negative
Case 15	Coccidioidomycosis	24	21	16.8 ng/*µ*L	N.a.	N.a.
Case 16	Coccidioidomycosis	24	14	107.5 ng/*µ*L	N.a.	N.a.
Case 17	Mycetoma/maduromycosis	18	13	26.3 ng/*µ*L	N.a.	N.a.
Control 1	Negative control	22	34	609.7 ng/*µ*L	N.a.	N.a.
Control 2	Negative control	23	25	655.3 ng/*µ*L	N.a.	N.a.
Control 3	Negative control	19	31	676.6 ng/*µ*L	N.a.	N.a.

**Table 3:** Comparison of histological and sequencing results. (Details on PCR results and sequencing quality can be found in supplementary materials 2–5.)

Sample I.D.	Histological diagnosis	PCR 1	PCR 2	PCR 3	PCR 4	PCR 5
Case 1	Chromoblastomycosis	Multiple species, no discrimination possible	Noninterpretable, poor/overlapping sequences	*Umbilicaria torrefacta/Placynthium nigrum *	Noninterpretable, poor/overlapping sequences	Noninterpretable, poor/overlapping sequences

Case 2	Mucormycosis	*Aspergillus* spp./*Penicillium* spp.	*Candida parapsilosis *	Noninterpretable, poor/overlapping sequences	Noninterpretable, poor/overlapping sequences	*Candida parapsilosis *

Case 3	Histoplasmosis	*Cryptococcus carnescens/Cryptococcus psychrotolerans/Cryptococcus peneaus/Taphrina maculans *	Noninterpretable, poor/overlapping sequences	Various *Umbilicaria* spp.	Various *Penicillium *spp.	—

Case 4	Mucormycosis	*Delitschia didyma *	Various *Candida* spp./*Yarrowia* spp./*Candida galli *	—	Multiple species, no discrimination possible/*Neofusicoccum* spp.	—

Case 5	Histoplasmosis or cryptococcosis	*Lactarius indigo*/*Candida piceae*/*Glomus* spp.	Noninterpretable, poor/overlapping sequences	Various *Umbilicaria* spp.	Noninterpretable, poor/overlapping sequences	Noninterpretable, poor/overlapping sequences

Case 6	Chromoblastomycosis	*Xylomyces* spp./*Speiropsis* spp./*Jahnula* spp./*Brachiosphaera* spp./*Megalohypha* spp./*Lactarius indigo Candida piceae*/*Glomus* spp.	Noninterpretable, poor/overlapping sequences	—	Noninterpretable, poor/overlapping sequences	—

Case 7	Rhinosporidiosis	Noninterpretable, poor/overlapping sequences	Noninterpretable, poor/overlapping sequences	Noninterpretable, poor/overlapping sequences	*Candida* spp./*Malassezia *spp./*Starmerella *spp.	—

Case 8	Mycetoma/maduromycosis	*Lactarius indigo/Candida piceae/Glomus* spp.	—	—	Noninterpretable, poor/overlapping sequences	—

Case 9	Rhinosporidiosis	Multiple species, no discrimination possible	Noninterpretable, poor/overlapping sequences	Various *Umbilicaria *spp.	*Rhizoctonia solani *	Noninterpretable, poor/overlapping sequences

Case 10	Mycetoma/maduromycosis	Multiple fungal species including *Penicillium* spp./*Aspergillus* spp.	Noninterpretable, poor/overlapping sequences	Noninterpretable, poor/overlapping sequences	Various *Penicillium* spp./*Talaromyces flavus *	Noninterpretable, poor/overlapping sequences

Case 11	Histoplasmosis	Noninterpretable, poor/overlapping sequences	*Candida parapsilosis *	Noninterpretable, poor/overlapping sequences	*Trametes* spp./*Tricholoma *spp./*Phellinus* spp.	—

Case 12	Histoplasmosis	*Candida parapsilosis *	*Candida parapsilosis *	*Candida parapsilosis *	*Candida parapsilosis *	*Candida parapsilosis/*Tremellalesspp./Saccharomycetales spp.

Case 13	Chromoblastomycosis	Multiple fungal species including *Penicillium* spp./*Aspergillus* spp.	*Candida parapsilosis/*various *Candida* spp.	*Penicillium *spp.	*Aspergillus *spp./*Ajellomyces capsulatus/Histoplasma capsulatum *	*Candida parapsilosis *

Case 14	Histoplasmosis	*Ajellomyces capsulatus/Histoplasma capsulatum *	Noninterpretable, poor/overlapping sequences	*Paecilomyces variotii *	*Ajellomyces capsulatus/ Histoplasmacapsulatum *	—

Case 15	Coccidioidomycosis	Multiple species, no discrimination possible	*Candida parapsilosis *	Noninterpretable, poor/overlapping sequences	*Candida parapsilosis *	*Candida parapsilosis *

Case 16	Coccidioidomycosis	*Delitschia didyma *	*Candida parapsilosis *	Noninterpretable, poor/overlapping sequences	Noninterpretable, poor/overlapping sequences	Noninterpretable, poor/overlapping sequences

Case 17	Mycetoma/maduromycosis	*Delitschia didyma *	Noninterpretable, poor/overlapping sequences	*Myceliophthora thermophila/Thielavia terrestris/Neurospora crassa *	*Madurella mycetomatis *	Noninterpretable, poor/overlapping sequences

Control 1	Negative control	*Funneliformis* spp./*Glomus* spp./*Candida salmanticensis *	Noninterpretable, poor/overlapping sequences	Various *Umbilicaria *spp.	*Davidiella tassiana *	—

Control 2	Negative control	Noninterpretable, poor/overlapping sequences	Noninterpretable, poor/overlapping sequences	Various *Umbilicaria *spp.	Noninterpretable, poor/overlapping sequences	—

Control 3	Negative control	Noninterpretable, poor/overlapping sequences	Noninterpretable, poor/overlapping sequences	*Umbilicaria *spp./*Lasallia* spp./*Boreoplaca ultrafrigida/Rhizoplaca huashanensis *	Noninterpretable, poor/overlapping sequences	—

## References

[1] Vandewoude K., Blot S., Benoit D., Depuydt P., Vogelaers D., Colardyn F. (2004). Invasive aspergillosis in critically ill patients: analysis of risk factors for acquisition and mortality. *Acta Clinica Belgica*.

[2] Castón-Osorio J. J., Rivero A., Torre-Cisneros J. (2008). Epidemiology of invasive fungal infection. *International Journal of Antimicrobial Agents*.

[3] Sangoi A. R., Rogers W. M., Longacre T. A., Montoya J. G., Baron E. J., Banaei N. (2009). Challenges and pitfalls of morphological identification of fungal infections in histologic and cytologic specimens. A ten-year retrospective review at a single institution. *The American Journal of Clinical Pathology*.

[4] Schwarz J. (1982). The diagnosis of deep mycoses by morphologic methods. *Human Pathology*.

[5] Watts J. C. (1994). Surgical pathology and the diagnosis of infectious diseases. *American Journal of Clinical Pathology*.

[6] Thankamani V., Dev L. (2012). *Rhinosporidium seeberi* proven as a fungus for the first time after a century since its discovery. *Research in Biotechnology*.

[7] Sewell D. L. (1995). Laboratory-associated infections and biosafety. *Clinical Microbiology Reviews*.

[8] Guarner J., Brandt M. E. (2011). Histopathologic diagnosis of fungal infections in the 21st century. *Clinical Microbiology Reviews*.

[9] Einsele H., Hebart H., Roller G. (1997). Detection and identification of fungal pathogens in blood by using molecular probes. *Journal of Clinical Microbiology*.

[10] Balajee S. A., Sigler L., Brandt M. E. (2007). DNA and the classical way: identification of medically important molds in the 21st century. *Medical Mycology*.

[11] Hata D. J., Buckwalter S. P., Pritt B. S., Roberts G. D., Wengenack N. L. (2008). Real-time PCR method for detection of zygomycetes. *Journal of Clinical Microbiology*.

[12] Khot P. D., Ko D. L., Hackman R. C., Fredricks D. N. (2008). Development and optimization of quantitative PCR for the diagnosis of invasive aspergillosis with bronchoalveolar lavage fluid. *BMC Infectious Diseases*.

[13] Hrncirova K., Lengerova M., Kocmanova I. (2010). Rapid detection and identification of mucormycetes from culture and tissue samples by use of high-resolution melt analysis. *Journal of Clinical Microbiology*.

[14] Hammond S. P., Bialek R., Milner D. A., Petschnigg E. M., Baden L. R., Marty F. M. (2011). Molecular methods to improve diagnosis and identification of mucormycosis. *Journal of Clinical Microbiology*.

[15] Fleischhacker M., Schulz S., Jöhrens K. (2012). Diagnosis of chronic disseminated candidosis from liver biopsies by a novel PCR in patients with haematological malignancies. *Clinical Microbiology and Infection*.

[16] White P. L., Barton R., Guiver M. (2006). A consensus on fungal polymerase chain reaction diagnosis?: a United Kingdom-Ireland evaluation of polymerase chain reaction methods for detection of systemic fungal infections. *Journal of Molecular Diagnostics*.

[17] Hagen R. M., Gauthier Y. P., Sprague L. D. (2002). Strategies for PCR based detection of *Burkholderia pseudomallei* DNA in paraffin wax embedded tissues. *Journal of Clinical Pathology—Molecular Pathology*.

[18] Quach N., Goodman M. F., Shibata D. (2004). In vitro mutation artifacts after formalin fixation and error prone translesion synthesis during PCR. *BMC Clinical Pathology*.

[19] Lu K., Ye W., Zhou L. (2010). Structural characterization of formaldehyde-induced cross-links between amino acids and deoxynucleosides and their oligomers. *Journal of the American Chemical Society*.

[20] Frickmann H., Tenner-Racz K., Eggert P. (2013). Influence of parasite density and sample storage time on the reliability of *Entamoeba histolytica*-specific PCR from formalin-fixed and paraffin-embedded tissues. *Diagnostic Molecular Pathology*.

[21] Henry T., Iwen P. C., Hinrichs S. H. (2000). Identification of *Aspergillus* species using internal transcribed spacer regions 1 and 2. *Journal of Clinical Microbiology*.

[22] Loeffler J., Henke N., Hebart H. (2000). Quantification of fungal DNA by using fluorescence resonance energy transfer and the light cycler system. *Journal of Clinical Microbiology*.

[23] Jordanides N. E., Allan E. K., McLintock L. A. (2005). A prospective study of real-time panfungal PCR for the early diagnosis of invasive fungal infection in haemato-oncology patients. *Bone Marrow Transplantation*.

[24] Knot P. D., Ko D. L., Fredricks D. N. (2009). Sequencing and analysis of fungal rRNA operons for development of broad-range fungal PCR assays. *Applied and Environmental Microbiology*.

[25] Dannaoui E., Schwarz P., Slany M. (2010). Molecular detection and identification of Zygomycetes species from paraffin-embedded tissues in a murine model of disseminated zygomycosis: a collaborative European Society of Clinical Microbiology and Infectious Diseases (ESCMID) Fungal Infection Study Group (EFISG) evaluation. *Journal of Clinical Microbiology*.

[26] Muñoz-Cadavid C., Rudd S., Zaki S. R. (2010). Improving molecular detection of fungal DNA in formalin-fixed paraffin-embedded tissues: comparison of five tissue DNA extraction methods using panfungal PCR. *Journal of Clinical Microbiology*.

[27] Rickerts V., Khot P. D., Myerson D., Ko D. L., Lambrecht E., Fredricks D. N. (2011). Comparison of quantitative real time PCR with sequencing and ribosomal RNA-FISH for the identification of fungi in formalin fixed, paraffin-embedded tissue specimens. *BMC Infectious Diseases*.

[28] Niesters H. G. M. (2001). Quantitation of viral load using real-time amplification techniques. *Methods*.

[29] Bialek R., Feucht A., Aepinus C. (2002). Evaluation of two nested PCR assays for detection of *Histoplasma capsulatum* DNA in human tissue. *Journal of Clinical Microbiology*.

[30] Bialek R., Konrad F., Kern J. (2005). PCR based identification and discrimination of agents of mucormycosis and aspergillosis in paraffin wax embedded tissue. *Journal of Clinical Pathology*.

[31] Clinical and Laboratory Standards Institute (2009). Interpretive criteria for identification of bacteria and fungi by DNA target sequencing. *Approved standard MM18-A, 1st Edition*.

[32] Jensen H. E., Aalbaek B., Schønheyder H. (1994). Immunohistochemical identification of aetiological agents of systemic bovine zygomycosis. *Journal of Comparative Pathology*.

[33] da Silva R. M., da Silva Neto J. R., Santos C. S. (2015). Fluorescent *in situ* hybridization of pre-incubated blood culture material for the rapid diagnosis of histoplasmosis. *Medical Mycology*.

