# Supplementary material for: Detection of Tropical Fungi in Formalin-Fixed, Paraffin-Embedded Tissue: Still an Indication for Microscopy in Times of Sequence-Based Diagnosis?
Source: Biomed Res Int. 2015 Apr 19;2015:938721. doi: 10.1155/2015/938721 (PMC4417575; doi:10.1155/2015/938721)
Supplement: Supplementary file 1 — Altogether, 6 supplementary materials are presented to provide further details regarding the applied methods and the observed results. Supplementary material 1 describes the applied pan-fungal PCR protocols, the inhibition control PCR and the sample quality assessment PCR in detail. The supplementary materials 2-6 are an addition to table 3 of the paper. They provide more detailed information regarding the observed ct-values, melting curve peaks (if applicable), detection of visible bands in electrophoresis (if applicable), readable sequence fragment lengths, coverage and sequence identities of pan-fungal PCRs 1-5. Thereby, they allow for a better interpretation of PCR and sequencing results. [file 938721.f1.pdf]

Supplementary Material 1: Applied PCR protocols adapted to the PCR cyclers used. N.a. = not applicable.

	Pan-fungal PCR 1	Pan-fungal PCR 2	Pan-fungal PCR 3	Pan-fungal PCR 4	Pan-fungal PCR 5	Inhibition control PCR	Sample quality assessment PCR
PCR cycler	LightCycler 2.0, Roche, Basel, Switzerland	Corbett RotorGene 6000, Qiagen, Hilden, Germany	Corbett RotorGene 6000, Qiagen	TProfessional Basis, BioMetra, An Analytik Jena Company	TProfessional Basis, BioMetra	Corbett RotorGene 6000, Qiagen	Corbett RotorGene 6000, Qiagen
Target	Variable 18S rRNA gene region (usually 482–503 base pair fragment)	Variable ITS-2 region of the fungal rRNA operon (usually 200–360 base pair fragment)	Variable 28S rRNA gene region (usually 330–350 base pair fragment)	Variable ITS-2 rRNA gene region, (usually 254 ± 43 base pair fragment)	Variable ITS-1 rRNA gene region (usually 295 ± 70 base pair fragment)	Phocid herpesvirus 1 (PhHV-1) 1 gB gene (89 base pair fragment)	Human 18S rRNA gene (155 base pair fragment)
PCR type	LightCycler real-time	EvaGreen real-time	EvaGreen real-time	Traditional PCR	Traditional PCR	TaqMan real-time	TaqMan real-time
Primers and probes							
Forward primer	RTP_1: 5′-ATT-GGA-GGG-CAA-GTC-TGG-TG-3′	5.8S: 5′-GTG-AAT-CAT-CGA-RTC-TTT-GAA-C-3′	28S-10: 5′-GAC-ATG-GGT-TAG-TCG-ATC-CTA-3′	ITS-3: 5′-GCA-TCG-ATG-AAG-AAC-GCA-GC-3′	ITS-1: 5′-TCC-GTA-GGT-GAA-CCT-GCG-G-3′	PhHV-267s 5′-GGG-CGA-ATC-ACA-GAT-TGA-ATC-3′	18S-f 5′-CTC-TTA-GCT-GAG-TGT-CCC-GC-3′

Reverse primer	RTP_2: 5′-CCG-ATC-CCT-AGT-CGG-CAT-AG-3′	28S1-rev: 5′-TAT-GCT-TAA-GTT-CAG-CGG-GTA-3′	28S-12-rev: 5′-CCT-TAT-CTA-CAT-TRT-TCT-ATC-AAC-3′	ITS-4: 5′-TCC-TCC-GCT-TAT-TGA-TAT-GC-3′	ITS-2: 5′-GCT-GCG-TTC-TTC-ATC-GAT-GC-3′	PhHV-337as 5′-GCG-GTT-CCA-AAC-GTA-CCA-A-3′	18S-r 5′-CTT-AAT-CAT-GGC-CTC-AGT-TCC-GA-3′
Probe(s)	RTP_p1: 5′-3FL-TTC-AAC-TAC-gAg-CTT-TTT-AAC-Tg-3′; RTP_p2: 5′-LC-Red640-AAC-AAC-TTT-AAT-ATA-CgC-TAT-Tgg A-3′	n.a.	n.a.	n.a.	n.a.	PhHV-305tq 5′-Cy55-TTT-TTA-TGT-GTC-CGC-CAC-CAT-CTG-GAT-C-BBQ-3′	18S-p 5′-FAM-CCG-AGC-CGC-CTG-GAT-ACC-GCA-GCT-A-TAMRA-3′
Adapted reaction chemistry							
Mastermix	Taq DNA polymerase in 10× PCR buffer (P1806 Sigma), (Sigma-Aldrich, St. Louis, MO, USA)	HotStarTaq master-mix (Qiagen), EvaGreen (Biotium, Hayward, CA)	HotStarTaq master-mix (Qiagen), EvaGreen (Biotium)	HotStarTaq master-mix (Qiagen)	HotStarTaq master-mix (Qiagen)	HotStarTaq master-mix (Qiagen)	HotStarTaq master-mix (Qiagen)
$MgCl_2$ (total)	3.0 mmol/l	3.0 mmol/l	3.0 mmol/l	2.5 mmol/l	2.5 mmol/l	5.0 mmol/l	6.0 mmol/l

Forward primer concentration	0.4 pmol/µl	0.6 pmol/µl	0.8 pmol/µl	0.2 pmol/µl	0.5 pmol/µl	4.0 pmol/µl	16 pmol/µl
Reverse primer concentration	0.4 pmol/µl	0.6 pmol/µl	0.8 pmol/µl	0.2 pmol/µl	0.5 pmol/µl	4.0 pmol/µl	16 pmol/µl
Probe(s) Concentration	0.5 pmol/µl	n.a.	n.a.	n.a.	n.a.	5.0 pmol/µl	4.5 pmol/µl
PCR protocol							
Initial denaturation	95°C, 10 min	95°C, 10 min	95°C, 10 min	94°C, 10 min	94°C, 10 min	95°C, 15 min	95°C, 10 min
Cycles	n=45	n=45	n=45	n=40	n=30	n=40	n=40
Denaturation	95°C, 15 s	95°C, 10 min	95°C, 10 min	94°C, 1 min	94°C, 30 s	95°C, 15	95°C, 15 s
Annealing	55°C, 10 s	55°C, 30 s	55°C, 30 s	51°C, 1 min	58°C, 30 s	60°C, 30 s	65°C, 65 s
Extension	72°C, 25 s	72°C, 40 s	72°C, 40 s	72°C, 1 min	72°C, 30 s	72°C, 30 s	65°C, 65 s
Final extension	-	-	-	72°C, 10 min	72°C, 30 s	-	-
Melting protocol							
Denaturation	95°C, 20 s	95°C, 2 min	95°C, 2 min	n.a.	n.a.	n.a.	n.a.
Annealing	45°C, 20 s	68°C, 30 s	68°C, 30 s	n.a.	n.a.	n.a.	n.a.
Melting	95°C, 0.2 °C/s	99°C, 0.2 C/s	99°C, 0.2 C/s	n.a.	n.a.	n.a.	n.a.
Cooling	40°C, 20 s	40°C, 20 s	40°C, 20 s	n.a.	n.a.	n.a.	n.a.

Supplementary Material 2: Results of pan-fungal PCR 1 (best matches). Ct = cycle threshold.

Sample I.D.	Histological diagnosis	Ct-value	Peak in the melting curve analysis	Readable sequence fragment length (base pairs)	Coverage	Identity	Sequence results (NCBI BLAST, organism: fungi)
Case 1	Chromoblastomycosis	–	74°C	503	100%	97%	Multiple species, no discrimination possible
Case 2	Mucormycosis	–	74°C	59	81%	96%	*Aspergillus* spp./*Penicillium* spp.
Case 3	Histoplasmosis	–	74°C	259	100%	97%	*Cryptococcus carnescens*/*Cryptococcus psychrotolerans*/*Cryptococcus peneaus/Taphrina maculans*
Case 4	Mucormycosis	–	86°C	23	100%	100%	*Delitschia didyma*
Case 5	Histoplasmosis or cryptococcosis	–	74°C	60	36%	100%	*Lactarius indigo*/*Candida piceae*/*Glomus* spp.
Case 6	Chromoblastomycosis	–	74°C	170	92%	94%	*Xylomyces* spp./*Speiropsis* spp./*Jahnula* spp./*Brachiosphaera*

							spp./*Megalohypha* spp.
				42	50%	100%	*Lactarius indigo Candida piceae*/*Glomus* spp.
Case 7	Rhinosporidiosis	–	73°C	–	–	–	Non-interpretable, poor/overlapping sequences
Case 8	Mycetoma/maduromycosis	–	74°C	56	37%	100%	*Lactarius indigo*/*Candida piceae*/*Glomus* spp.
Case 9	Rhinosporidiosis	–	74°C	237	100%	97%	Multiple species, no discrimination possible
Case 10	Mycetoma/maduromycosis	38	74°C	521	98%	98%	Multiple fungal species including *Penicillium* spp./*Aspergillus* spp.
Case 11	Histoplasmosis	–	74°C	–	–	–	Non-interpretable, poor/overlapping sequences
Case 12	Histoplasmosis	33	74°C	463	100%	100%	*Candida parapsilosis*
Case 13	Chromoblastomycosis	–	74°C	527	100%	99%	Multiple fungal species including *Penicillium* spp./*Aspergillus* spp.
Case 14	Histoplasmosis	–	74°C	269	71%	92%	*Ajellomyces capsulatus*/*Histoplasma capsulatum*
Case 15	Coccidioidomycosis	–	74°C	298	100%	91%	Multiple species, no discrimination possible

Case 16	Coccidioidomycosis	–	75°C	39	50%	100%	*Delitschia didyma*
Case 17	Mycetoma/madurомycosis	–	80°C	46	50%	100%	*Delitschia didyma*
Control 1	Negative control	–	74°C	20	100%	100%	*Funneliformis* spp./*Glomus* spp./*Candida salmanticensis*
Control 2	Negative control	–	74°C	–	–	–	Non-interpretable, poor/overlapping sequences
Control 3	Negative control	–	74°C	–	–	–	Non-interpretable, poor/overlapping sequences

Supplementary Material 3: Results of pan-fungal PCR 2 (best matches). Ct = cycle threshold.

Sample I.D.	Histological diagnosis	Ct-value	Peak in the melting curve analysis	Readable sequence fragment length (base pairs)	Coverage	Identity	Sequence results (NCBI BLAST, organism: fungi)
Case 1	Chromoblastomycosis	26	87°C	–	–	–	Non-interpretable, poor/overlapping sequences
Case 2	Mucormycosis	25	87°C	130	99%	98%	*Candida parapsilosis*
				114	100%	100%	*Candida parapsilosis*
Case 3	Histoplasmosis	31	89°C	–	–	–	Non-interpretable, poor/overlapping sequences
Case 4	Mucormycosis	32	Multiple	68	100%	97%	Various *Candida* spp./*Yarowia* spp.
				66	100%	98%	*Candida galli*
Case 5	Histoplasmosis or cryptococcosis	29	89°C	–	–	–	Non-interpretable, poor/overlapping sequences
Case 6	Chromoblastomycosis	43	Multiple	–	–	–	Non-interpretable, poor/overlapping sequences
Case 7	Rhinosporidiosis	30	77°C	–	–	–	Non-interpretable, poor/overlapping sequences
Case 8	Mycetoma/Madur	–	–	–	–	–	–

	omycosis						
Case 9	Rhinosporidiosis	25	87°C	–	–	–	Non-interpretable, poor/overlapping sequences
Case 10	Mycetoma/madur omycosis	25	87°C	–	–	–	Non-interpretable, poor/overlapping sequences
Case 11	Histoplasmosis	32	86°C	141	99%	100%	*Candida parapsilosis*
				130	99%	98%	*Candida parapsilosis*
Case 12	Histoplasmosis	17	87°C	231	99%	100%	*Candida parapsilosis*
Case 13	Chromoblastomy cosis	23	87°C	238	65%	100%	*Candida parapsilosis*
				161	54%	97%	Various *Candida* spp.
Case 14	Histoplasmosis	–	86°C	–	–	–	Non-interpretable, poor/overlapping sequences
Case 15	Coccidioidomyco sis	27	86°C	240	100%	99%	*Candida parapsilosis*
Case 16	Coccidioidomyco sis	29	86°C	113	100%	99%	*Candida parapsilosis*
Case 17	Mycetoma/madur omycosis	30	87°C	–	–	–	Non-interpretable, poor/overlapping sequences
Control 1	Negative control	–	89°C	–	–	–	Non-interpretable, poor/overlapping sequences
Control 2	Negative control	–	89°C	–	–	–	Non-interpretable, poor/overlapping sequences
Control 3	Negative control	–	88°C	–	–	–	Non-interpretable,

Supplementary Material 4: Results of pan-fungal PCR 3 (best matches). Ct = cycle threshold.

Sample I.D.	Histological diagnosis	Ct–value	Peak in the melting curve analysis	Readable sequence fragment length (base pairs)	Coverage	Identity	Sequence results (NCBI BLAST, organism: fungi)
Case 1	Chromoblastomycosis	33	Multiple	137	100%	96%	*Umbilicaria torrefacta*/*Placynthium nigrum*
Case 2	Mucormycosis	32	88°C	–	–	–	Non-interpretable, poor/overlapping sequences
Case 3	Histoplasmosis	40	91°C	139	100%	99%	Various *Umbilicaria* spp.
Case 4	Mucormycosis	–	–	–	–	–	–
Case 5	Histoplasmosis or cryptococcosis	39	Multiple	151	100%	99%	Various *Umbilicaria* spp.
Case 6	Chromoblastomycosis	–	–	–	–	–	–
Case 7	Rhinosporidiosis	43	Multiple	–	–	–	Non-interpretable, poor/overlapping sequences
Case 8	Mycetoma/madurомycosis	–	–	–	–	–	–
Case 9	Rhinosporidiosis	33	91°C	273	71%	96%	Various *Umbilicaria* spp.

Case 10	Mycetoma/madur omycosis	30	91°C	–	–	–	Non-interpretable, poor/overlapping sequences
Case 11	Histoplasmosis	–	–	–	–	–	Non-interpretable, poor/overlapping sequences
Case 12	Histoplasmosis	26	88°C	370	99%	100%	*Candida parapsilosis*
Case 13	Chromoblastomy cosis	31	91°C	147	100%	98%	*Penicillium* spp.
Case 14	Histoplasmosis	–	85°C	357	100%	98%	*Paecilomyces variotii*
Case 15	Coccidioidomyco sis	37	88°C	–	–	–	Non-interpretable, poor/overlapping sequences
Case 16	Coccidioidomyco sis	41	91°C	–	–	–	Non-interpretable, poor/overlapping sequences
Case 17	Mycetoma/madur omycosis	44	Multiple	374	99%	98%	*Myceliophthora thermophila*/*Thielavia terrestris*/*Neurospora crassa*
Control 1	Negative control	–	–	123	100%	96%	Various *Umbilicaria* spp.
Control 2	Negative control	–	–	123	100%	98%	Various *Umbilicaria* spp.
Control 3	Negative control	–	–	120	100%	98%	*Umbilicaria* spp./*Lasallia* spp./*Boreoplaca ultrafrigida*/*Rhizoplaca huashanensis*

Supplementary Material 5: Results of pan-fungal PCR 4 (best matches). Ct = cycle threshold.

Sample I.D.	Histological diagnosis	PCR positive (visible bands in electrophoresis)	Readable sequence fragment length (base pairs)	Coverage	Identity	Sequence results (NCBI BLAST, organism: fungi)
Case 1	Chromoblastomycosis	Positive	–	–	–	Non-interpretable, poor/overlapping sequences
Case 2	Mucormycosis	Positive	–	–	–	Non-interpretable, poor/overlapping sequences
Case 3	Histoplasmosis	Positive	502	65%	97%	various *Penicillium* spp.
Case 4	Mucormycosis	Positive	439	76%	99%	multiple species, no discrimination possible
Case 5	Histoplasmosis or Cryptococcosis	Positive	403<br>16	28%<br>–	99%<br>–	*Neofusicoccum* spp.<br>Non-interpretable, poor/overlapping sequences
Case 6	Chromoblastomycosis	Positive	–	–	–	Non-interpretable, poor/overlapping sequences
Case 7	Rhinosporidiosis	Positive	45	93%	100%	*Candida* spp./*Malassezia* spp./*Starmerella* spp.
Case 8	Mycetoma/madur omycosis	Positive	–	–	–	Non-interpretable, poor/overlapping sequences
Case 9	Rhinosporidiosis	Positive	205	83%	85%	*Rhizoctonia solani*

Case 10	Mycetoma/maduromycosis	Positive	241	100%	99%	various Penicillium spp.
			146	83%	95%	*Talaromyces flavus*
Case 11	Histoplasmosis	Positive	305	100%	97%	*Trametes* spp./*Tricholoma* spp./*Phellinus* spp.
			142	100%	99%	*Trametes* spp.
Case 12	Histoplasmosis	Positive	493	68%	100%	*Candida parapsilosis*
Case 13	Chromoblastomycosis	Positive	72	100%	97%	*Aspergillus* spp.
			20	100%	100%	*Ajellomyces capsulatus*/*Histoplasma capsulatum*
Case 14	Histoplasmosis	Positive	286	100%	100%	*Ajellomyces capsulatus*/*Histoplasma capsulatum*
Case 15	Coccidioidomycosis	Positive	103	100%	98%	*Candida parapsilosis*
Case 16	Coccidioidomycosis	Positive	–	–	–	Non-interpretable, poor/overlapping sequences
Case 17	Mycetoma/maduromycosis	Positive	444	82%	100%	*Madurella mycetomatis*
Control 1	Negative control	Positive	401	79%	94%	*Davidiella tassiani*
Control 2	Negative control	Positive	–	–	–	Non-interpretable, poor/overlapping sequences
Control 3	Negative control	Positive	–	–	–	Non-interpretable, poor/overlapping sequences

Supplementary Material 6: Results of pan-fungal PCR 5 (best matches). Ct = cycle threshold.

Sample I.D.	Histological diagnosis	PCR positive (visible bands in electrophoresis)	Readable sequence fragment length (base pairs)	Coverage	Identity	Sequence results (NCBI BLAST, organism: fungi)
Case 1	Chromoblastomy cosis	Positive	–	–	–	Non-interpretable, poor/overlapping sequences
Case 2	Mucormycosis	Positive	66	100%	100%	*Candida parapsilosis*
Case 3	Histoplasmosis	–	–	–	–	–
Case 4	Mucormycosis	–	–	–	–	–
Case 5	Histoplasmosis or Cryptococcosis	Positive	–	–	–	Non-interpretable, poor/overlapping sequences
Case 6	Chromoblastomy cosis	–	–	–	–	–
Case 7	Rhinosporidiosis	–	–	–	–	–
Case 8	Mycetoma/Madur omycosis	–	–	–	–	–
Case 9	Rhinosporidiosis	Positive	–	–	–	Non-interpretable, poor/overlapping sequences
Case 10	Mycetoma/madur omycosis	Positive	–	–	–	Non-interpretable, poor/overlapping sequences
Case 11	Histoplasmosis	–	–	–	–	–

Case 12	Histoplasmosis	Positive	452	98%	100%	*Candida parapsilosis*/*Tremellales* spp./*Saccharomycetales* spp.
Case 13	Chromoblastomycosis	Positive	128	81%	100%	*Candida parapsilosis*
Case 14	Histoplasmosis	–	–	–	–	–
Case 15	Coccidioidomycosis	Positive	227	100%	100%	*Candida parapsilosis*
Case 16	Coccidioidomycosis	Positive	–	–	–	Non-interpretable, poor/overlapping sequences
Case 17	Mycetoma/maduromycosis	Positive	–	–	–	Non-interpretable, poor/overlapping sequences
Control 1	Negative control	–	–	–	–	–
Control 2	Negative control	–	–	–	–	–
Control 3	Negative control	–	–	–	–	–
